# Soybean Meal Can Be Replaced by Faba Beans, Pumpkin Seed Cake, Spirulina or Be Completely Omitted in a Forage-Based Diet for Fattening Bulls to Achieve Comparable Performance, Carcass and Meat Quality

**DOI:** 10.3390/ani11061588

**Published:** 2021-05-28

**Authors:** Magdalena Keller, Beat Reidy, Andreas Scheurer, Lukas Eggerschwiler, Isabelle Morel, Katrin Giller

**Affiliations:** 1 ETH Zurich, Institute of Agricultural Sciences, Universitaetstrasse 2, 8092 Zurich, Switzerland; magdalena.keller@usys.ethz.ch; 2 School of Agricultural, Forest and Food Sciences HAFL, Bern University of Applied Sciences, 3052 Zollikofen, Switzerland; beat.reidy@bfh.ch (B.R.); andreas.scheurer@bfh.ch (A.S.); 3 Agroscope, Route de la Tioleyre 4, 1725 Posieux, Switzerland; lukas.eggerschwiler@agroscope.admin.ch (L.E.); isabelle.morel@agroscope.admin.ch (I.M.)

**Keywords:** beef cattle, alternative protein sources, fatty acids, grass silage, sustainable production systems, microalgae

## Abstract

**Simple Summary:**

The sustainability of intensive beef production systems can be greatly improved using domestic protein sources as alternatives to imported soybean meal. Potential alternatives include indigenous grain legumes such as faba beans, food industry by-products including locally available oil cakes, or novel protein sources such as microalgae. Moreover, diets based on grassland-derived feeds increase dietary protein supply and have the potential to enhance food system sustainability. This study aimed to assess the effects on fattening performance, carcass and meat quality, and the meat fatty acid profile of beef cattle when replacing, or omitting, soybean meal in the diet. Bulls fed a grass/maize-silage based diet supplemented with concentrate containing either soybean meal, faba beans, pumpkin seed cake, or spirulina had similar growth performances and carcass and meat quality. No differences were observed in the meat fatty acid profiles. Most notably, omitting soybean meal without additional protein-concentrate replacements resulted in comparable carcass and meat quality without affecting the meat fatty acid profile while maintaining a high-performance level. Consequently, the grass silage-based diet supplemented with a grain-based concentrate without additional protein source was the most sustainable diet for growing bulls tested in this study.

**Abstract:**

The aim of the study was to investigate the complete substitution of imported soybean meal in beef cattle diets and the consequences on performance, meat, and adipose tissue quality. Thirty growing crossbred Limousin bulls, with an initial bodyweight of 164 ± 13 kg and 4.3 ± 0.3 months of age, were fed a grass/maize-silage based diet with little additional concentrate (0.5:0.3:0.2). Concentrates contained either soybean meal (positive control), faba beans, pumpkin seed cake, or spirulina (*Arthrospira platensis*), resulting in about 226 g crude protein (CP)/kg concentrate dry matter (DM) and 158 g CP/kg total diet DM. A grain-based concentrate providing just 135 g CP/kg concentrate DM and 139 g CP/total diet DM served as a negative control. Bulls of all groups had comparable average daily gains (1.43 ± 0.1 kg) and feed intakes (6.92 ± 0.37 kg). Carcass and meat quality did not differ among groups. The fatty acid profile of meat lipids was hardly affected. These results indicate that soybean meal can be replaced by any of the tested protein sources without impairing performance or meat quality. Importantly, bulls fed the negative control achieved a fattening and slaughter performance comparable to that of the protein-supplemented groups without affecting meat and adipose tissue quality. Thus, the present findings suggest that feeding crossbred bulls a grass/maize-silage based diet does not require additional protein supplementation.

## 1. Introduction

Intensive beef production in Europe relies heavily on maize silage and large amounts of concentrate to achieve high average daily gains (ADG) and a desirable carcass quality. While these diets are rich in energy and starch, they necessitate the addition of extra protein to meet dietary requirements of beef cattle. Protein requirements, particularly those of metabolizable protein, are traditionally met by the concentrate portion of the diet. In Swiss intensive beef production, concentrate proportions amount to almost one third of the total diet on a dry matter (DM) basis (300 g/kg DM) [1]. Imported soybean meal (SBM) is often used as the main protein source for beef cattle as it is widely available and richer in valuable rumen undegradable protein (RUP) than most other protein sources. However, soy cultivation is increasingly controversial due to its negative environmental impacts (e.g., monocropping, loss of biodiversity and natural habitats, long transport routes, and high input requirements including land, fertilizers, and fuel) and poor socioeconomics (e.g., rural depopulation, loss of employment due to high levels of mechanization and agrarian transformation) [2,3].

In contrast, grassland-derived feeds, that increase protein supply when included as part of the basal diet, are rarely used due to their comparably lower energy contents and a perceived lower animal performance [4,5]. Previous findings indicate that no additional protein may be required when feeding high-quality grass silage to growing bulls supplemented with barley-based concentrates to maintain performance, carcass, and meat quality [6,7,8]. However, average body weight (BW) gain was rather low (1.05 kg per day), whereas the dietary concentrate proportions applied were fairly high (300–700 g/kg DM) [6,7,9]. Limiting the dietary concentrate proportion might increase production responses to protein supplements [8,10]. Therefore, when aiming for a high growth performance with ADG > 1.30 kg during limited concentrate and high forage supply, sources specifically providing metabolizable protein are likely required due to the typically high ruminal protein degradability (RPD) of grassland-derived feeds.

Promising protein sources as alternatives to imported SBM include other grain legumes such as faba beans (*Vicia faba*). They can be grown globally [11,12] and improve the sustainability of cropping systems by preserving soil fertility and introducing beneficial pre-crop effects [12,13]. The high crude protein (CP) and starch contents as well as the high RPD of the faba bean make it a suitable protein source for cattle, particularly when combined with low-protein forages like maize silage [14,15,16]. However, the effects of faba beans as a replacement for SBM in grass-silage based diets for beef cattle have not yet been investigated.

Aside from grain legumes, locally available oil cakes present an interesting alternative protein source for the replacement of SBM in beef cattle diets. In comparison to rather common rapeseed and sunflower oil cakes, pumpkin seed cake has an outstandingly high CP content, around 600 g/kg DM [17]. However, information about its nutritional value and suitability for beef cattle diets is scarce. Antunović, et al. [18] and Klir et al. [19] published the first promising results in lambs and dairy goats, respectively.

A third protein source that may be an interesting alternative to SBM are protein-rich microalgae such as spirulina (*Arthrospira platensis,* 730 g CP/kg DM [20]). Phototrophic spirulina can be cultivated in open ponds or closed photo bioreactors [21] on non-arable land such as rooftops [22] and at a high water-use efficiency [23]. When compared to soybean and rapeseed, spirulina provides a protein yield 15 to 20 times greater per unit area [23,24]. Due to these properties, spirulina has gained an interest as a protein replacement for SBM in cattle diets. However, previous studies investigating the use of spirulina as a dietary bovine protein source have focused on dairy cattle [25,26,27], while data on beef cattle are missing. The relatively high proportion of the somewhat rare fatty acid (FA), C18:3 *n*–6 (γ-linolenic acid), in spirulina may yield a meat FA profile different to that of SBM fed animals, which has not yet been investigated in beef cattle. To our knowledge, comparisons of these protein sources in beef cattle fed a grass/maize-silage based diet have not been published.

Therefore, the aim of this study was to investigate the effects of a complete replacement of SBM by faba beans, pumpkin seed cake, and spirulina, or its omission, on animal performance, meat, and adipose tissue quality in beef cattle fed a high proportion of grass silage accompanied by a limited amount of concentrate (200 g/kg total diet DM). The following hypotheses were tested: (i) Some or all of these protein sources can replace SBM without adverse effects on growth and slaughter performance. (ii) The diet without additional protein sources impairs animal performance due to insufficient metabolizable protein supply. (iii) Replacing SBM does not affect meat quality. (iv) The intake of spirulina with its characteristic FA profile compared to the other protein sources results in a specific meat FA profile.

## 2. Animals, Materials, and Methods

### 2.1. Animals and Diets

The experiment was conducted at the AgroVet-Strickhof research station (Lindau, Switzerland) and was approved by the Cantonal Veterinary office of Zurich, Switzerland (license no. ZH129/18). It comprised five diets differing in concentrate protein source. The positive control (diet SB) contained solvent extracted SBM (Landi, Sense-Düdingen, Switzerland), which was replaced by either faba beans (fenaco Genossenschaft, Getreide, Ölsaaten, Futtermittel, Berne, Switzerland; diet FB), cold pressed pumpkin seed cake (Ölmühle Garting, Schnaitsee, Germany; diet PS) or the microalga, spirulina (Institut für Getreideverarbeitung, Nuthetal, Germany; diet SP) (Table 1). A concentrate with no additional protein source (diet NP) served as a negative control. The concentrates PS and NP were pelleted, while the concentrates SB, FB and SP were provided as meal due to unexpected technical problems in the pelleting process.

The positive control diet was designed according to the Swiss feeding recommendation for beef cattle with an ADG of 1.30 kg [28]. Based on this calculation, the concentrates containing the tested protein sources were calculated to provide a target CP content of 230 g/kg DM by combining the protein sources with varying amounts of wheat, maize grain, wheat bran, molasses, and animal fat (Table 2). However, the requirements for metabolizable protein, particularly for metabolizable protein deriving from ruminal available energy (APDE) were deficient by 28, 23, and 18% in the early fattening period of 150, 200, and 250 kg BW, respectively. The target CP content for the NP diet was intended to be 130 g CP/kg DM.

Thirty Limousin-sired crossbred bulls (dams: Brown Swiss, 14; Holstein, 5; Swiss Fleckvieh, 5; Original Brown Swiss, 3; Red Holstein, 3) with an initial age of 4.3 ± 0.3 months (mean ± standard deviation) and an initial BW of 164 ± 13 kg were used. Animals were vaccinated as calves with Rispoval RS + PI3 intranasal (Zoetis, Dublin Ireland) prior to entering the feeding experiment but were not dewormed. Experimental groups were created by balancing for initial BW to achieve a comparable average BW among groups. Despite the differing quantities, the variation in the sires (*n* = 15) and the five dam breeds was accounted for to avoid creating treatment groups of animals sired by only one bull or born to only one breed. The experimental groups were then randomly allocated to one of the five experimental diets (*n* = 6).

Bulls were housed in three pens, of ten bulls each, in a free-stall barn with freely accessible, designated areas for the animals to feed, run (with access to an outside area) and lie down. Straw was used as bedding, with fresh straw added three times per week. Two animals of each dietary treatment were represented in each pen. Each animal had exclusive access to its own feeding trough by electronically operated gates and transponder collars (Waagen Doehrn, Wesel, Germany).

All animals received a diet similarly composed of grass silage, maize silage, and concentrate in a ratio of 0.5:0.3:0.2 on DM basis. Two different batches of wilted grass silage were fed, the first from experimental onset until experimental week 26, and the second from experimental week 27 until the end of the experiment. Both grass silages were obtained from a ryegrass-clover ley (predominantly ryegrass) and harvested at the beginning of ear emergence of the ryegrass in two consecutive years. The first batch was harvested as third and fourth cut and was stored in a bunker silo. The second batch was harvested as fourth cut and preserved in large round bales. Two different batches of whole plant maize silage were used. Both were harvested at half milkline stage and stored in bunker silos that were changed in week 11. No silage additives were used.

The grass and maize silage mixture was freshly prepared every second day. It was offered daily in the morning at *ad libitum* access. The forage was supplemented with 12 g/kg DM of a mineral–vitamin mixture providing, per kg, calcium, 138 g, phosphorus, 46 g, magnesium, 36 g, sodium, 167 g, zinc, 5 g, manganese, 3 g, copper, 1 g, vitamin A, 625,000 IE, vitamin D_3_, 62,500 IE, vitamin E, 1 mg, and *Saccharomyces cerevisiae* (*NCYC Sc* 47), 333 colony forming units. Additionally, animals had free access to a NaCl-containing licking block. The vitamin–mineral mix was added directly to the forage mixture instead of the experimental concentrates to ensure appropriate allocation of the minerals and vitamins to animals from the beginning of the experiment. At this time point, it was not known whether the palatability of the concentrates and consequently mineral intakes would be impaired. The concentrates were top-dressed by hand twice daily, or three times if the animals were offered >2 kg concentrate/day.

One week prior to the start of the experiment, animals were gradually adapted to the experimental diets. From the second experimental week onwards, individual feed intake was recorded throughout the experimental period on two consecutive days of each week by weighing the amount of forage provided and the leftovers after 24 h. In order to maintain the designated silage:concentrate ratio of 0.8:0.2 for each animal throughout the fattening period, individual concentrate allocation was adjusted to measured *ad libitum* silage intake every 2 weeks.

The BW was measured every second week using a cattle scale (Ixonix FX 15, Texas Trading GmbH, Windach, Germany). Bulls were weighed in the afternoon and were not fasted before weighing.

### 2.2. Slaughter, Carcass Quality, and Sampling

The target live weight for slaughter was set at 520 kg following the common practice for Terra-Swiss labelled beef. Before slaughter, the animals were fasted overnight for about 12 h and transported within 45 min to the University of Zurich’s abattoir (Zurich, Switzerland). Slaughters were performed on four different dates within a 62-day period. Animals that achieved a BW near the target weight were selected and grouped, resulting in groups of six, ten, eight, and six bulls slaughtered on the four dates, respectively. On two slaughter dates, at least one animal of each feeding group was slaughtered, whereas on the two other slaughter dates bulls from four of the five treatments were slaughtered. At slaughter, the animals’ age averaged 12.6 ± 0.7 months. Animals were slaughtered every 35 min in a random order concerning experimental groups. Stunning using a captive bolt gun was followed by exsanguination. Hot carcass weight was recorded within 30 min after stunning. Dressing percentage was calculated as the percentage of hot carcass weight to BW the afternoon before slaughtering. Carcasses were graded according to the Swiss classification system CH-TAX [29] for conformation (C = excellent, X = poor) and fatness score (1 = too lean, 3 = optimal and homogenous fat cover, 5 = excessively fat) by an independent professional. This system is comparable with the EUROP grading scheme.

Samples of the *Musculus longissimus thoracis et lumborum* (LTL) were excised 3 h after stunning from the left carcass side between the 10th thoracic and the 5th lumbar vertebra. A slice of 2 cm was cut from the caudal side and trimmed of residual adipose and connective tissue. After homogenization in a household blender (Moulinette type DP-700, Moulinex, Ecully, France), the meat was vacuum packed and stored at −20 °C. The remaining sample was cut into two evenly sized pieces, which were stored uncovered at 4 °C for 24 h. At 24 h *post mortem (p.m.)*, muscle samples were gently blotted dry with paper towels, weighed, vacuum packed, and aged for 7 (caudal side) or 21 (cranial side) days at 4 °C.

Heart, liver, spleen, kidney, and perirenal fat were immediately removed from the carcasses and their weight was recorded. Samples of subcutaneous fat were obtained within 30 min after stunning from the back of the left carcass, homogenized (Moulinette type DP-700, Moulinex, Ecully, France), vacuum packed and stored at −20 °C.

### 2.3. Feed Sampling and Proximate Analysis of the Feeds

Samples of grass and maize silage were collected 15 and 11 times, respectively, from both batches of silage to account for possible silage variations. Concentrates were sampled three times as each concentrate was derived from a single batch. The protein sources were derived from one batch each and were sampled once, prior to mixing into the concentrates. The feed refusals were sampled and their composition was analyzed occasionally during the experiment. Since no or only minor differences were observed between the proximate composition of the leftovers and the forage mixture provided, the composition of the leftovers was not further considered. The forage samples were dried at 60 °C for 48 h prior to milling. Forages and concentrates were ground in a centrifugal mill (ZM 200, Retsch GmbH, Haan, Germany) to pass a 1-mm sieve before analysis.

All individual feed samples were analyzed for their proximate composition with standard methods [30,31]. Dry matter and total ash were determined using a thermos gravimetric device model (TGS 701, Leco Corporation, St. Josephs, MI, USA; AOAC index no. 942.05). Nitrogen (N) was assessed using a C/N-analyzer (TruMac CN, Leco Corporation; AOAC index no. 968.06). The CP was calculated as 6.25 × N. Ether extract (EE) was analyzed in a Soxhlet extractor (extraction system B-811, Buechi, Flawil, Switzerland; AOAC index no. 963.15). Ether extract in spirulina and the spirulina-containing concentrate was determined after HCl hydrolysis (Hydrolysis Unit B-425, Buechi, Flawil, Switzerland). Ash-corrected neutral detergent fiber (NDF; with heat-stable α-amylase from Sigma-Aldrich, St. Louis, MO, USA) and acid detergent fiber (ADF) in feed were measured using the Fibertherm FT 12 (Art. 13-0026, Gerhardt GmbH & Co. KG, Koenigswinter, Germany; VDLUFA methods 6.5.1 and 6.5.2, respectively). Fiber fractions could not be analyzed in spirulina and SP concentrate due to poor reliability and repeatability of results caused by the extremely fine powder form of the spirulina. Acid detergent lignin (ADL; VDLUFA method 6.5.3) was analyzed in forages by incubation with sulphuric acid (72%) for 3 h subsequent to ADF analysis. Protein fractions were determined by standardized procedures [32]. Non-protein N (NPN; fraction A) was calculated as the difference between total CP and true protein precipitated using tungstic acid. The true protein was distinguished by its solubility in borate-phosphate buffer, neutral, and acid detergent solutions into fractions B_1_, B_2,_ and B_3_, respectively. The true protein insoluble in acid detergent is represented in the C-fraction. In spirulina these fractions could not be distinguished for the same reasons as described for fiber analysis. Instead, the true protein content of spirulina was analyzed (VDLUFA method 4.4.1).

Metabolizable energy (ME) contents of the forages, SBM, and faba beans were estimated based on proximate nutrient composition and digestibility coefficients according to Agroscope [33]. The ME content of pumpkin seed cake was estimated based on linseed cake (80–110 g EE/kg DM) due to missing digestibility coefficients for pumpkin seed cake [33]. For spirulina, ME content was estimated as 0.0159 × digestible CP (g/kg DM) + 0.0377 × digestible EE (g/kg DM) + 0.0147 × digestible N-free extract (g/kg DM) [28]. The N-free extract (g/kg DM) was calculated as DM (g/kg wet weight) − (ash (g/kg DM) + EE (g/kg DM) + CP (g/kg DM)). Digestibility coefficients for CP, EE, and N-free extract were 0.738, 0.625, and, 0.67, respectively [34]. Values for metabolizable protein resulting from RUP and microbial protein synthesized from ruminal available energy (APDE) and from RUP and microbial protein synthesized from ruminal available nitrogen (APDN) [28] of the forages, SBM and faba bean were estimated according to Agroscope [33]. Metabolizable protein contents of pumpkin seed cake and spirulina could not be estimated due to missing data on ruminal protein degradability.

### 2.4. Analysis of Meat Quality and Fat Shelf Life

The chemical composition of homogenized meat was analyzed as described for the feed samples. For intramuscular fat, hydrolysis by HCl was performed before extraction as described by Mueller et al. [35]. Temperature and pH of the excised LTL sample were measured 24 h *p.m.* with a pH meter with integrated temperature sensor (testo 205, Testo Ltd., Alton, Hampshire, UK). After ageing for 7 or 21 days, the meat was blotted dry and reweighed to determine ageing loss. From the aged samples, two 1-cm thick slices were dissected and allowed to bloom (freshly cut side facing up) for 1 h in the dark at 4 °C. At five points, color was measured using a Chroma Meter (model CR-300 with light source C, D_65_; Konica Minolta, Tokyo, Japan) in the CIE L* a* b* (lightness, redness, and yellowness, respectively) color space. Another four 2-cm thick slices were obtained. Both pairs were weighed, one pair was placed individually in a two-layer net and hung in a sealed plastic bag for 24 h at 4 °C to determine drip loss [36]. To assess cooking loss, the other two slices were vacuum packed and cooked in a water bath at 75 °C until they reached a core temperature of 72 °C. The latter was controlled with a digital food thermometer (testo 108, Testo Ltd., Alton). The cooked samples were cooled in cold tap water for 10 min, blotted dry and reweighed. Afterwards, seven to ten cylindrical cores with a diameter of 1.27 cm were cut from the cooked meat samples in parallel to the muscle fibers with a cork borer. Shear force was measured perpendicular to the muscle fibers on the cores by using a Warner-Bratzler shear force blade attached to a texture analyzer (model ProLine table-top machine Z005, Zwick Roell, Ulm, Germany). Average values per animal were calculated for each variable for statistical analysis.

The homogenized subcutaneous fat was melted at 80 °C for 60 min and sieved through a kitchen sieve to remove connective tissue before determining the oxidative stability with a Rancimat (model 697, Metrohm, Herisau, Switzerland) at 110 °C and an airflow of 20 L/h.

### 2.5. Fatty acid Analysis in Feeds and Meat

Fatty acid profiles of feed samples and homogenized meat samples were analyzed as outlined by Wolf, et al. [37]. Briefly, total lipids were extracted using hexane:isopropanol (HIP) in a ratio of 3:2 (vol/vol) and FA were converted to FA methyl esters (FAME) under cooking conditions using methanolic NaOH and BF_3_ [38], followed by gas chromatographic analysis (HP 6890, Agilent Technologies, Inc., Wilmington, PA, USA). Dietary lipids were extracted using an accelerated solvent extractor (ASE 200, Dionex Coporation, Sunnyvale, CA, USA), while muscle lipids were extracted directly by dispersion in HIP (Polytron^®^ model PT 6000, Kinematica AG, Luzern, Switzerland). Prior to adding methylation reagents, C11:0 triglyceride (Fluka Chemie, Buchs, Switzerland) was added as internal standard. Separation of FAME was performed by injecting 1 µL of FAME at a split ratio of 1:20 onto a CP7421 column (wall-coated open tubular fused silica 200 mm × 0.25 mm; Varian Inc., Lake Forest, CA, USA). Compounds were carried by hydrogen at a flow rate of 1.7 mL/min. Detector temperature was 270 °C. The detailed temperature program is described in Wolf, et al. [37]. Peaks were characterized by comparing the retention times to a 37-component FAME standard (Sigma Aldrich, Steinheim, Germany). Peak areas of FAME were quantified using the HP ChemStation^®^ software (version Rev. B.04.03-SP1; Agilent, Palo Alto, CA, USA). Proportions of FA were expressed as percentage of the total area of FAME injected. Following Ulbricht and Southgate [39], the atherogenicity [(C12:0 + 4 × C14:0 + C16:0)/(ΣMUFA + Σ*n*–6 + Σ*n*–3)] and thrombogenicity [(C14:0 + C16:0 + C18:0)/(0.5 × ΣMUFA + 0.5 × Σ*n*–6 + 3 × Σ*n*–3 + (Σ*n*–6/Σ*n*–3))] of the intramuscular fat were calculated (MUFA: monounsaturated FA).

### 2.6. Statistical Analysis

Data were analyzed with Rstudio (version 1.2.5001; Rstudio, Inc., Boston, MA, USA) using the aov function to assess diet effect on animal-performance related variables. Initial BW was included in the model as a covariate for days on experimental feeds and concentrate DM intake (DMI). This variable was not considered in any other performance-related variable as it was found to have no effect. A mixed model analysis using the lmer function was applied for carcass, some meat (pH_24 h *post mortem*_, temperature_24 h *post mortem*_, chemical composition of fresh meat), and fat quality (oxidative stability) data as well as for FA data considering diet as fixed and slaughter day as random effect as not all animals were slaughtered on the same day. Animal was considered as the experimental unit. Post hoc analysis was done by applying the glht function to test for differences between diets. Data of meat ash content was inverted for statistical analysis. The Box Cox transformation was used to calculate the best λ to transform data of age at start of the experiment, meat ash content, C14:1, C16:1, C17:1, C18:2 *trans*-11, *cis*-15, C18:2 *n–*6 *cis*, C18:3 *n–*6, C18:3 *n–*3, C20:4 *n–*6, C20:5 *n–*3, C22:4 *n–*6, C22:5 *n–*3, C22:6 *n–*3, sum of polyunsaturated FA (PUFA), sum of *n–*3 FA, sum of *n–*6 FA, and *n*–6/*n*–3 FA ratio. For meat quality data obtained after the two different ageing periods, a mixed model for repeated measures with diet, ageing period and their interaction as fixed effects and slaughter day and animal as random effects was used. A post-hoc test was done by calculating contrast using the glht function to estimate the significance of differences among diets within days of ageing and within diets among days of ageing. Shear force data was square transformed prior to analysis. Results of untransformed data are presented as arithmetic means ± standard error of the mean (SEM). Effects at *p* < 0.05 were considered statistically significant. *P*-values of 0.05 < *p* < 0.1 were considered a trend.

## 3. Results

### 3.1. Feed Composition, Growth and Slaughter Performance

The protein sources differed substantially in CP content (Table 1), which had to be balanced differently by other concentrate ingredients (Table 2). The NP concentrate provided 135 g CP/kg DM, whereas all other concentrates had an average CP content of 226 ± 5.0 g/kg DM. The complete diets containing the different protein sources were widely isonitrogenous and isoenergetic (Table 3). Depending on the combination of grass- and maize silage batches used (Table 2), they contained on average 157 ± 1.1 g CP/kg and 9.7 ± 0.06 MJ ME/kg DM (Table 3). The SB-diet was on average richer in APDE and APDN than the FB-diet. The NP-diet provided on average 9.7 ± 0.10 MJ ME/kg DM and 139 ± 2.3 g CP/kg DM with an even more pronounced deficiency (36, 32 and 28% at 150, 200, 250 kg BW) in the metabolizable protein supply than the positive control diet.

The protein sources also differed regarding their proportions of protein fractions (Table 1). While most of the protein of SBM and pumpkin seed cake belonged to the B_2_-fraction, a high proportion of the B_1_-fraction was found in the faba beans. Faba bean CP had the highest proportion of fraction A.

The FA profile of spirulina differed from that of the other protein sources. The major FA found in spirulina were C16:0, C18:3 *n*–6, and C18:2 *n*–6 *cis* (descending order). In SBM, faba beans, and pumpkin seed cake, the predominant FA were C18:2 *n*–6 *cis,* C18:1 *cis*-9, and C16:0 (descending order), whereas no or only traces of C18:3 *n*–6 were detected. Spirulina contained more than double the amount of total saturated FA (SFA) compared to the other protein sources but only small amounts of monounsaturated FA (MUFA). Soybean meal provided the highest proportion of *n*–3 FA and the lowest *n–*6*/n*–3 FA ratio, which was greatest in spirulina. However, the major FA found in all concentrates were identical (C18:2 *n*–6 *cis*, C18:1 *cis*-9, and C16:0) but present in varying proportions (Table 2).

The average fattening period (days) as well as initial and slaughter BW (Table 4) were similar for all groups. No differences in BW were found among groups during the experimental period (data not shown). Treatment groups showed similar growth performances at any given time point and ADG did not differ among groups (1.43 ± 0.018 kg across all groups). No differences were observed in DMI for total, silage, or concentrate intakes among groups, thus the feed conversion ratios (DMI/kg ADG) were similar. Concentrate provided at each meal was always completely consumed. Slaughter performance was not affected by diet. On average, all bulls had a carcass weight of 279 ± 2.0 kg and a dressing percentage of 53.9 ± 0.22 %. Carcasses received similar and favorable conformation scores at fairly low fat cover scores (Table 4). No significant dietary effects were observed for proportionate organ weights or perirenal fat.

### 3.2. Physicochemical Meat Quality

At 24 h *p.m.*, pH and temperature in excised LTL samples were similar among diets (Table 5). Muscles from all groups had similar contents of moisture, protein, intramuscular fat, and ash. Diet had no effect on the oxidative stability of the subcutaneous fat. Ageing loss increased with ageing time (*p* = 0.003) across all groups but was only prevalent (*p* = 0.005) in SP. Drip loss remained unaffected by diet and ageing period, while cooking loss tended (*p* = 0.075) to differ between diets. Meat color variables did not differ among diets but varied (all *p* < 0.001) with ageing period. Overall, lightness increased with time but within experimental groups, this effect reached significance only in SB (*p* = 0.009) and PS (*p* = 0.005). After 21 days of ageing, the beef from SB, FB, PS, and NP was more red than after 7 days of ageing (*p* = 0.018; *p* = 0.026; *p* < 0.001; *p* = 0.005, respectively). Yellowness was more intense after 21 days compared to 7 days of ageing in SB (*p* = 0.002), PS (*p* = 0.007), and NP (*p* = 0.002), whereas in FB it tended to vary (*p* = 0.088). Shear force was similar between groups but values were lower (*p* < 0.001) after 21 compared to 7 days of ageing. In variables where ageing was investigated, no significant dietary interactions occurred. 

### 3.3. Fatty Acid Profile of Intramuscular Fat

Proportions of the major FA were comparable for all groups (Table 6), with an average of 32.6 ± 0.60, 23.6 ± 0.29, and 15.3 ± 0.31 g/100 g FA for C18:1 *cis*-9, C16:0, and C18:0, respectively. Dietary effects were found for C16:0 *iso* (*p* = 0.031) and C18:1 *cis*-12 (*p* = 0.015). The SB meat had a higher (*p* = 0.026) proportion of C16:0 *iso* compared to PS meat. The C18:1 *cis*-12 proportion was higher for PS than for SB and SP meat (*p* = 0.049 and *p* = 0.047, respectively). Proportions of C18:1 *trans*-11 tended to differ among groups (*p* = 0.058). Diet had no significant effects on any other FA. However, when numerically comparing proportions of C18:3 *n*–3, C20:4 *n*–6, C20:5 *n*–3, and C22:5 *n*–3, they were 45%, 27%, 36%, and 20% higher in NP than SB, respectively. Classes of SFA, MUFA, and PUFA as well as *n*–6 and *n*–3 FA remained unaffected. A significant difference among groups was found for the *n*–6/*n*–3 FA ratio, which was lower for FB compared to PS (*p* < 0.001) and NP (*p* = 0.008), and for SP compared to PS (*p* = 0.008). Neither the atherogenicity nor thrombogenicity index was affected by the diet.

## 4. Discussion

### 4.1. Characteristics of Protein Sources Tested

Tested protein sources differed in CP content; however, values were within the ranges previously reported in the literature for each protein source [11,17,20,40,41]. The numerical variations in the CP fractions, however, reflect likely differences in protein solubility and thus indicate differences in RPD [32]. The high proportion of the B_2_-fraction of SBM refers to a low RPD, whereas the numerically high proportions of the A and B_1_-fractions in faba beans indicate a rather high RPD, which is consistent with former reports [41,42]. The comparably high proportion of the B_2_-fraction in the pumpkin seed cake used in the present study indicates a moderate RPD, ranging between that of faba beans and SBM. However, to provide a better estimation of the alternative protein sources’ nutritional value for ruminants, particularly for pumpkin seed cake and spirulina, further investigations regarding the RPD using different batches of these protein sources are required to perform statistical analysis and validate the present results.

No meaningful fractionation was possible for spirulina due to its powdery form. Costa, et al. [43] reported a higher in vitro protein degradability for spirulina than for SBM, whereas Wild, et al. [44] found a limited in vitro protein fermentation, indicating a high proportion of RUP in spirulina but a low intestinal digestibility. This requires further clarification. In general, differences in the RPD between different protein sources had no effect on animal performance, carcass, and meat quality traits in the present study. This may differ in maize-silage based diets, which is generally limited in metabolizable protein.

### 4.2. Growth and Slaughter Performance

The absence of effects on intake, growth, and carcass quality, when replacing SBM with faba beans, support the results of previous studies with Simmental bulls fed a maize-silage based diet [16] and Marchigiana bulls fed a diet with > 550 g concentrate/kg total diet DM [45]. Cutrignelli, et al. [45] observed a lower BW at an earlier fattening period for animals fed faba beans instead of SBM, possibly due to limited RUP supply. However, in the present study, no differences were found in growth performance at any given time point. Despite these converse results, faba beans seem to be an appropriate replacement for SBM in the diets of fattening bulls. Puhakka, et al. [46] observed a reduction in silage and total DMI when including faba beans in the diets of dairy cattle resulting in a decreased milk and milk protein yield. This contrasted with the present intake findings. High palatability is a prerequisite for an alternative protein source. Contrasting findings suggest that investigating the palatability of faba beans compared to SBM in a larger animal study may be of interest.

Similar to the present study, the suitability of pumpkin seed cake as a replacement for SBM was confirmed by Antunović, et al. [18] in lambs (similar carcass quality with partial SBM replacement) and by Klir, et al. [19] in dairy goats when SBM was completely substituted (no change in milk yield). These results coincide with the present outcomes and suggest that pumpkin seed cake has a high palatability and indeed presents an interesting alternative to SBM in the nutrition of different ruminant species.

In contrast to the present findings, the inclusion of spirulina in dairy cow diets reduced DMI [47] or concentrate intake when mixed with spirulina [26,48]. This discrepancy in results indicates that the addition of pure spirulina powder (mixed with water) to the total mixed ration or concentrate proportion, in the mentioned studies, likely decreased the palatability of the respective feed. In the present study, spirulina was pre-mixed with all concentrate ingredients, this avoided potential palatability problems. Likewise, Manzocchi, et al. [27] did not observe a reduction in DMI when mixing spirulina with molasses into a hay-based diet fed to dairy cows. Importantly, experimental concentrates applied in the present study were always completely consumed by the animals, indicating a high palatability of these concentrates independent of protein source and presentation (pelleted vs. meal).

The similar animal performance, even when omitting additional protein sources, likely resulted from the comparably higher dietary proportion of grass silage than typically included in conventional diets for fattening bulls in Switzerland that consist primarily of maize silage [1]. Additionally, the CP content (about 175 g/kg DM) of the grass silage used in the present experiment was fairly high compared to the usual range of 100 to 160 g/kg DM [14]. Studies in growing cattle fed *ad libitum* high-quality grass silage and grain-based concentrates showed that an additional protein supply had no or a limited overall effect on the growth performance and carcass quality [6,7,8,10], indicating a sustained microbial protein synthesis.

In contrast, decreasing the amount of concentrate in grass-silage based diets may limit microbial protein synthesis due to limited ruminal available energy, thus increasing the likelihood of a greater response to RUP supply [8,10]. This may be particularly relevant during the early growth period in which the requirements for metabolizable protein, in relation to metabolizable energy, are higher [28]. However, the postulated metabolizable protein requirements were not sufficiently met by the SB-diet in the present study, particularly in the early growth period. The deficiency in metabolizable protein in relation to the postulated requirements was most pronounced in the NP-diet. Therefore, it was even more surprising that the omission of a protein supplement did not impair growth performance. Thus, it can be assumed that the supply of metabolizable protein was already sufficiently met by the forage as well as the grain proportion of the concentrate or that metabolizable energy was the more limiting factor. The latter is unlikely as the average ADG of 1.43 kg in the present study was almost as high as that reported by Staerfl, et al. [49] for Limousin bulls fattened on a common intensive maize silage/concentrate beef cattle diet. Consequently, the present results indicate that the metabolizable protein requirements for growing crossbred Limousin bulls are likely overestimated. This requires further investigation, also regarding other commonly used beef cattle breeds.

Unexpectedly, all groups showed a higher growth performance than initially predicted. This inconsistency could be explained by a higher muscle (protein) retention potential than anticipated in the feeding recommendations, which provide an average recommendation for various breeds.

Despite the higher growth performance observed in the present study, the target fat cover score of 3 was not met by any of the experimental diets, while dressing percentage and carcass conformation were satisfactory in all groups and comparable to results of Staerfl et al. [49]. Carcasses graded with lower fat cover scores are usually less desirable for retail which may significantly reduce the profit margin when farmers are paid for carcass quality traits. Extending the fattening period may have resulted in better fat cover scores but would have increased final BW and thus carcass weight. Heavier carcasses do not comply to the desired standards of the Swiss beef market and likewise lead to a reduction in the profit margin. Additionally, a longer fattening period may have decreased meat tenderness due to increased age at slaughter [50]. Improving these aspects potentially outweighs the lower fat cover.

Subcutaneous fat deposition mainly depends on energy supply, which is crucial during the late fattening period [51]. As the diet composition was not modified over the course of the present experiment, the lower proportions of concentrate and maize silage compared to conventional beef cattle diets were clearly limited in energy supply during late fattening. Further investigations are required to assess the optimal time point to adjust the energy supply for an optimal fat cover score while maintaining the highest possible amount of grass silage in the diet.

### 4.3. Meat Quality

The physicochemical meat quality was not affected by the diets, including that without protein supplementation. In contrast, Cutrignelli, et al. [52] found a reduced water holding capacity in meat of faba-bean fed Marchigiana bulls and Calabrò et al. [15] observed a slight reduction in intramuscular fat content in meat of buffalo bulls fed faba beans instead of SBM. The partial substitution of SBM by pumpkin seed cake fed to fattening lambs did not affect meat color [18], which supports the present results obtained with a complete replacement of SBM by pumpkin seed cake. It also seems that omitting protein supplementation (here rapeseed meal) in grass-silage and barley-based diets is without consequence for meat composition, water holding capacity, meat color, and shear force of beef cattle [8] which is comparable to the present study.

The prolongation of ageing time from 1 to 3 weeks, following common Swiss practice, significantly affected some of the meat quality traits. Ageing loss was increased by 21%, especially in SP but this from a generally low level. Numerical values are consistent with findings reported by Li et al. [53] for beef aged in vacuum for 8 or 19 days. Lightness, redness, and yellowness increased with prolonged ageing within SB and PS, while in NP, lightness was not affected and in FB, only redness was affected. This partly disagrees with Li, et al. [53], who only found an ageing time effect on lightness, but not on red- or yellowness in vacuum aged beef. On the other hand, color changes due to ageing have been previously reported for vacuum aged beef by Boakye and Mittal [54]. However, the meat color remained unaffected by ageing in SP, which may be attributed to the high content of antioxidant carotenoids in spirulina possibly transferred to the muscle tissue [21,55]. Prolonging ageing successfully promoted tenderization, as exhibited from the lower shear force and reported repeatedly by others [56,57].

### 4.4. Fatty Acid Profile of the Intramuscular Fat

Few and minor changes were found in the FA profile (namely in C16:0 *iso*, C18:1 *cis*-12, and, in tendency, in C18:1 *trans*-11) of the intramuscular fat in response to the different diets. Although the concentrates SB, PS, and NP provided numerically higher proportions of PUFA than FB and SP, the PUFA proportion in the LTL remained unaffected. The overall moderate fat content in the concentrates as well as biohydrogenation of unsaturated FA in the rumen may have contributed to this finding. Compared to meat of bulls fed a conventional diet based on maize and concentrate [15,58,59], generally higher proportions of PUFA and *n*–3 FA and a favorably lower *n*–6/*n*–3 FA ratio (1.9 to 2.2) in meat lipids were observed across all groups in the present study. This can be explained by the higher amount of grass silage fed in the present study, which provided higher dietary proportions of PUFA, particularly *n*–3 FA, to the animals and consequently improved the *n*–6/*n*–3 FA ratio compared to maize-silage based diets [49,58]. From the human nutrition perspective, a high intake of PUFA with an *n*–6/*n*–3 FA ratio of ≤ 4:1, is considered beneficial for maintaining health and reducing the risk of cardiovascular diseases [60]. The ratio of *n*–6/*n*–3 FA was favorably reduced by the FB compared to the PS diet, even though this difference was slight. Considering the low-fat content of the beef, the improved *n*–6/*n*–3 FA ratio would likely have a limited relevance regarding the meat nutritional value for human consumption.

Surprisingly, feeding of spirulina with its particularly deviating FA profile had no clear effect on the FA profile of intramuscular fat, including C18:3 *n*–6, which was highly prevalent in spirulina. Kashani et al. [61] supplemented dual-purpose lambs with spirulina and reported slightly lower C18:3 *n*–6 proportions in their microalga material compared to that used in the present study (17.2 vs. 26.7 g/100 g FA). For the lambs’ *Longissimus dorsi* muscle, no proportion of C18:3 *n*–6 was reported in the presentation of the FA profile, leading to the assumption that this characteristic FA was not detected and/or there were no differences to report. In contrast, when spirulina was fed to dairy cows [27], the milk fat showed higher proportions of C18:3 *n*–6, even though only to a small extent. The differences in the transfer of C18:3 *n*–6 to milk [27] and muscle tissue (present study) cannot be explained with certainty. It is probable that differences in ruminal biohydrogenation and generally different FA transport efficiencies, to milk and meat, played a role in this finding.

## 5. Conclusions

Based on the present findings, SBM can be replaced by any of the tested protein sources on an isonitrogenous basis in diets comprising 50% grass silage, 30% maize silage, and 20% concentrate without impairing performance, thus confirming hypothesis (i). Nevertheless, this may differ in typical maize-silage based fattening diets with particularly limited metabolizable protein supply. Additional studies are required to evaluate the potential of these protein sources to replace SBM in such diets. Notably, omitting additional dietary protein resulted in a comparable fattening and slaughter performance, disproving hypothesis (ii). It can be concluded that the combination of grass and maize silage and a wheat-based concentrate provided enough metabolizable protein for Limousin crossbred bulls with an ADG of about 1.43 kg or that the bulls’ growth potential was higher but compromised due to other factors, for instance dietary energy. As none of the diets affected carcass and meat quality, hypothesis (iii) was confirmed. Hypothesis (iv) was disproved as no difference in meat FA profiles were observed.

In summary, the high dietary proportion of grass silage improved the meat FA profile compared to values reported for conventional fattening diets, while maintaining reasonable animal performance and carcass and meat quality, without additional metabolizable protein-concentrate supplementation. This is good news from an environmental perspective, as omitting additional protein likely lowers urinary and fecal nitrogen excretion, and thus, limits ammonia emission. Moreover, if a grass-silage based diet does not require additional protein supplements, the independence from imported SBM is enhanced and high costs to produce alternative protein sources, particularly spirulina, are avoided.

## Figures and Tables

**Table 1:** Chemical composition and selected fatty acids (FA) of tested protein sources.

Protein Source	Soybean Meal	Faba Beans	Pumpkin Seed Cake	Spirulina
Proximate contents (g/kg DM)				
DM (g/kg wet weight)	882	864	925	945
Organic matter	932	954	889	917
Crude protein	536	275	623	710
Ether extract	16.1	21.6	90.1	67.6
Neutral detergent fiber	131	398	139	n.a.
Acid detergent fiber	94	198	111	n.a.
Gross energy (MJ/kg DM)	19.6	18.5	20.7	22.1
Crude protein fractions (g/100 g crude protein) ^1^				
A	8.3	25.1	10.1	9.4
B_1_	6.5	43.6	19.6	91.6
B_2_	82.1	17.5	68.7	
B_3_	2.5	12.5	1.1	
C	0.74	1.20	0.68	
FA (g/100 g total FA)				
C12:0	0.12	2.46	0.02	0.17
C14:0	0.16	0.69	0.12	0.11
C16:0	15.6	13.5	13.2	44.4
C16 *iso*	0.05	0.09	0.03	1.43
C16:1 *n*–7	0.15	0.39	0.15	3.37
C17:0	0.16	0.16	0.05	0.13
C16:2	0.00	0.00	0.00	0.31
C17:1	0.07	0.04	0.02	0.16
C18:0	3.96	2.76	4.95	1.05
C16:4	0.00	0.00	0.00	0.24
C18:1 *trans*-11	0.00	0.00	0.00	0.00
C18:1 *cis*-9	18.7	28.5	29.60	1.68
C18:1 *cis-*11	1.79	0.36	0.82	0.31
C18:1 *cis*-12	0.00	0.00	0.00	0.00
C18:2 *n*–6 *cis*	51.0	45.0	49.0	18.6
C18:3 *n*–6	0.00	0.04	0.00	26.66
C18:3 *n*–3	6.37	2.95	0.63	0.05
C20:0	0.29	1.05	0.39	0.06
C20:1 *n*–9	0.23	0.51	0.11	0.06
C20:2 *n*–6	0.07	0.11	0.08	0.25
C20:4 *n*–6	0.00	0.00	0.00	0.07
C22:0	0.35	0.43	0.18	0.00
C20:5 *n*–3	0.06	0.04	0.09	0.03
C23:0	0.11	0.12	0.06	0.05
C22:2	0.19	0.06	0.15	0.00
C24:0	0.23	0.21	0.15	0.03
∑ Saturated FA	21.2	21.8	19.2	47.6
∑ Monounsaturated FA	21.1	30.0	30.8	5.9
∑ Polyunsaturated FA	57.7	48.2	50.0	46.5
∑ *n*–3	6.43	2.99	0.73	0.08
∑ *n*–6	51.0	45.2	49.1	45.9
*n*–*6/n*–3 FA	8	15	67	574

DM: dry matter; n.a.: not analyzed; ^1^ A: non-protein nitrogen (NPN); B_1_: true soluble protein; B_2_: neutral detergent (ND) soluble protein; B_3_: ND insoluble true protein, soluble in acid detergent (AD), C: insoluble in AD.

**Table 2:** Components of the experimental concentrates, chemical composition and selected fatty acids (FA) of concentrates and forages.

Feed	Grass Silage		Maize Silage		Concentrates				
	1–26 ^1^	27–40 ^1^	1–10 ^1^	11–40 ^1^	SB	FB	PS	SP	NP
*n*	10	5	2	9	3	3	3	3	3
Ingredients (g/kg DM)									
Soybean meal					277	-	-	-	-
Faba beans					-	748	-	-	-
Pumpkin seed cake					-	-	227	-	-
Spirulina powder					-	-	-	198	-
Wheat grain					450	171	500	516	450
Maize grain					237	45	248	250	225
Wheat bran					11	-	-	-	300
Molasses					20	20	20	20	20
Tallow-lard mixture					5	16	5	16	5
Proximate contents (g/kg DM)									
DM (g/kg wet weight)	360	558	397	318	882	870	882	881	878
Organic matter	864	876	966	962	968	964	961	971	967
Crude protein	183	168	69	81	228	238	225	214	135
Ether extract	37.0	26.1	31.3	29.4	26.0	31.4	48.6	47.4	34.7
Neutral detergent fiber	477	512	414	471	464	473	459	n.a.	461
Acid detergent fiber	344	333	270	311	105	137	93	n.a.	128
Acid detergent lignin	50.2	56.4	44.2	39.5	n.a.	n.a.	n.a.	n.a.	n.a.
Gross energy (MJ/kg DM)	17.6	17.9	18.2	18.4	18.6	18.5	18.8	19.0	18.4
Metabolizable energy (MJ/ kg DM)	8.3	7.8	10.3	10.0	13.5	12.5	13.5	13.3	12.8
APDE (g/kg DM)	67	78	61	62	155	109	- ^2^	- ^2^	105
APDN (g/kg DM)	115	80	41	50	171	153	- ^2^	- ^2^	94
FA (g/100 g total FA)									
*n*	3	3	2	3	2	2	2	2	2
C14:0	0.66	0.50	0.18	0.22	0.55	1.35	0.49	1.02	0.50
C16:0	15.0	16.2	13.2	13.1	16.6	19.7	15.7	24.6	16.6
C16:0 *iso*	1.96	1.85	0.13	0.17	0.09	0.14	0.06	0.37	0.11
C16:1 *n*-7	0.23	0.22	0.17	0.18	0.56	1.30	0.49	1.62	0.52
C17:0	0.14	0.16	0.11	0.13	0.22	0.46	0.18	0.36	0.21
C18:0	1.15	1.43	2.11	2.04	4.77	9.54	5.40	7.23	3.98
C18:1 *trans*-11	0.00	0.00	0.00	0.00	0.19	0.50	0.16	0.39	0.17
C18:1 *cis*-9	1.8	2.4	24.6	23.9	24.9	30.8	27.6	23.2	22.9
C18:1 *cis*-11	0.43	0.40	0.64	0.66	1.07	1.04	0.96	1.00	0.95
C18:1 *cis*-12	0.00	0.00	0.00	0.00	0.03	0.06	0.02	0.04	0.02
C18:2 *n*–6 *cis*	13.2	15.1	52.4	50.0	45.8	29.6	45.0	32.3	48.5
C18:3 *n*–6	0.23	0.14	0.04	0.05	0.02	0.03	0.02	2.52	0.03
C18:3 *n*–3	61.2	57.9	3.4	6.7	2.8	2.0	1.6	2.6	3.0
C20:0	0.36	0.57	0.61	0.51	0.29	0.57	0.33	0.23	0.25
C20:1 *n*–9	0.36	0.22	0.31	0.37	0.45	0.49	0.33	0.39	0.54
C20:4 *n*–6	0.00	0.00	0.00	0.00	0.02	0.03	0.01	0.03	0.02
C22:0	0.78	0.72	0.36	0.32	0.18	0.23	0.16	0.10	0.16
C20:5 *n*–3	0.04	0.09	0.11	0.06	0.04	0.02	0.12	0.10	0.10
C24:0	0.66	0.60	0.50	0.52	0.17	0.13	0.15	0.10	0.17
∑ Saturated FA	21.4	22.8	17.5	17.5	23.2	32.7	22.8	34.3	22.2
∑ Monounsaturated FA	3.6	3.8	26.3	25.5	28.0	35.5	30.2	27.8	25.9
∑ Polyunsaturated FA	75.0	73.4	56.3	57.0	48.8	31.8	47.0	37.9	51.9
∑ *n*–3 FA	61.4	58.0	3.6	6.8	2.8	2.0	1.7	2.7	3.1
∑ *n*–6 FA	13.6	15.3	52.5	50.1	45.9	29.8	45.1	35.0	48.7
*n*–6/*n*–3 FA	0.2	0.3	14.6	7.4	16.4	14.9	26.5	13.0	15.7

APDE: metabolizable protein derived from ruminal available energy; APDN: metabolizable protein derived from ruminal protein fermentation; DM: dry matter; FB: faba beans; GS: grass silage; MS: maize silage; n.a.: not analyzed; NP: no additional protein source; PS: pumpkin seed cake; SB: soybean meal; SP: spirulina. ^1^ Periods of experimental weeks fed. ^2^ metabolizable protein content of pumpkin seed cake and spirulina unknown.

**Table 3:** Average proximate composition of complete experimental diets containing soybean meal (SB), faba beans (FB), pumpkin seed cake (PS), spirulina (SP), or no additional protein source (NP).

Proximate Contents (g/kg DM)	Complete Diets				
	SB	FB	PS	SP	NP
Organic matter	916	916	915	917	916
Crude protein	158	160	157	155	139
Ether extract	30.8	31.8	35.3	35.0	32.5
Neutral detergent fiber	475	476	474	n.a.	474
Acid detergent fiber	281	288	279	279	286
Gross energy (MJ/kg DM)	18.1	18.1	18.1	18.2	18.0
Metabolizable energy (MJ/kg DM)	9.8	9.6	9.8	9.8	9.7
APDE (g/kg DM)	85	76	-	-	75
APDN (g/kg DM)	100	96	-	-	85

APDE: metabolizable protein derived from ruminal available energy; APDN: metabolizable protein derived from ruminal protein fermentation; n.a.: not analyzed.

**Table 4:** Feed intake, growth, and slaughter performance of fattening bulls fed a grass/maize-silage based diet complemented with concentrates containing soybean meal (SB), faba beans (FB), pumpkin seed cake (PS), spirulina (SP), or no additional protein source (NP) (*n* = 6 per group).

Concentrate	SB	FB	PS	SP	NP	SEM	*p*-Value
Average fattening period (days)	245	246	253	253	245	11.5	0.837
Age at start (months) ^1^	4.4	4.4	4.4	4.2	4.3	0.22	0.860
Body weight (kg)							
Initial (start of experiment)	164	165	161	164	166	8.28	0.971
At slaughter	518	522	524	518	510	5.82	0.428
Average weight gain (kg/day)	1.45	1.46	1.44	1.40	1.41	0.047	0.771
DM intake (DMI; kg/day)							
Total	6.79	6.85	7.02	6.96	6.98	0.217	0.845
Silage	5.43	5.50	5.60	5.56	5.58	0.178	0.872
Concentrate	1.36	1.35	1.42	1.40	1.40	0.045	0.647
Feed conversion ratio (kg DMI/kg gain)	4.69	4.69	4.88	4.94	4.98	0.207	0.467
Hot carcass weight (kg)	278	279	287	278	276	5.26	0.318
Dressing percentage	53.7	53.4	54.8	53.6	54.0	0.59	0.532
Conformation score ^2^	3.7	3.9	4.4	3.7	3.7	0.30	0.220
Fat cover score ^3^	2.4	1.7	2.3	1.8	2.3	0.44	0.406
Organ weights (g/kg carcass weight)							
Heart	6.58	6.80	6.64	7.00	7.03	0.301	0.279
Liver	21.6	22.2	21.0	20.7	20.8	0.68	0.106
Spleen	3.77	3.52	3.26	3.86	3.44	0.292	0.078
Kidneys	3.75	3.76	3.49	3.61	3.78	0.650	0.491
Perirenal fat (g/kg carcass weight)	14.0	13.3	15.3	17.6	12.1	3.12	0.183

n.s.: not significant; SEM: standard error of the mean. ^1^ Data were transformed for statistical analysis but means of nontransformed data are presented in the table. ^2^ Defined as 1 = poor and 5 = excellent according to CH-TAX classification. ^3^ Defined as 1 = too lean, 3 = optimum, evenly covered with fat, 5 = excessively fat according to CH-TAX classification.

**Table 5:** Physicochemical quality of the *longissimus thoracis et lumborum* aged for 7 or 21 days from fattening bulls fed a grass/maize-silage based diet complemented with concentrates containing various protein sources or no additional protein source (*n* = 6 per group).

Concentrate (C)	SB		FB		PS		SP		NP		SEM	*p*-Values		
pH_24 h *postmortem*_	5.63		5.67		5.81		5.75		5.64		0.109	0.515		
Temperature_24 h *postmortem*_ (°C)	5.68		5.62		5.37		5.57		5.38		0.15	0.475		
Chemical composition (g/kg)														
Moisture	739		754		748		751		750		4.9	0.127		
Protein	232		223		222		225		228		3.7	0.141		
Fat (Ether extract)	8.91		8.27		8.42		8.01		8.45		2.047	0.995		
Ash ^1^	12.3		13.2		12.4		12.3		14.3		0.93	0.076		
Oxidative stability (h)	4.12		4.14		3.49		4.68		4.6		1.28	0.641		
**Ageing Period in Days (A)**	**7**	**21**	**7**	**21**	**7**	**21**	**7**	**21**	**7**	**21**		**C**	**A**	**C × A**
Water holding capacity (%)														
Aging loss	1.33	1.61	1.26	1.40	1.29	1.35 ^2^	1.37^a^	1.95^b^	1.41	1.72 ^2^	0.258	0.510	0.003	0.377
Drip loss	1.14	1.04	0.97	0.96	0.79	0.94	1.02	1.08	0.93	0.890	0.181	0.625	0.850	0.798
Cooking loss	23.7	24.8	24.0	23.4	22.9	19.2 ^2^	25.7	25.9	24.9	22.0	2.58	0.075	0.244	0.495
Color														
L* (lightness)	39.8 ^a^	42.0 ^b^	40.7	41.9	38.0 ^a^	40.3 ^b^	40.5	40.8	40.1	41.6	1.26	0.298	<0.001	0.266
a* (redness)	15.6 ^a^	16.7 ^b^	15.9 ^a^	16.9 ^b^	14.2 ^a^	16.2 ^b^	15.3	15.8	15.3 ^a^	16.6 ^b^	0.97	0.667	<0.001	0.092
b* (yellowness)	11.6 ^a^	13.6 ^b^	12.2	13.6	10.7 ^a^	12.5 ^b^	12.0	12.6	11.3 ^a^	13.4 ^b^	0.7	0.418	<0.001	0.356
Shear force (N) ^1^	100.9 ^b^	77.4 ^a^	89.3 ^b^	64.7 ^a^	97.6 ^b^	69.8 ^a 2^	98.0 ^b^	70.7 ^a^	100.4 ^b^	72.7 ^a^	7.68	0.505	<0.001	0.805

FB: faba beans; NP: no additional protein source; PS: pumpkin seed cake; SB: soybean meal; SEM: standard error of the mean; SP: spirulina. ^1^ Data were transformed for statistical analysis but means of nontransformed data are presented in the table. ^2^ Means calculated with *n* = 5. ^a,b^ Means carrying different superscripts within variable and feeding group are different at *p* < 0.05.

**Table 6:** Fatty acid (FA) profile of the *longissimus thoracis et lumborum* of fattening bulls fed a grass/maize-silage based diet complemented with concentrates containing soybean meal (SB), faba beans (FB), pumpkin seed cake (PS), spirulina (SP), or no additional protein source (NP), (*n* = 6 per group).

FA (g/100 g Total FA) ^1^	SB	FB	PS	SP	NP	SEM	*p*-Value
C14:0	2.10	1.70	1.73	1.88	1.84	0.217	0.866
C14:1 ^2^	0.48	0.39	0.38	0.36	0.37	0.151	0.871
C15:0	0.44	0.43	0.43	0.43	0.44	0.030	0.984
C15:0 *iso*	0.21	0.20	0.19	0.20	0.21	0.015	0.641
C16:0	24.1	23.4	23.7	23.9	23.1	0.94	0.838
C16:0 *iso*	0.21 ^b^	0.20 ^ab^	0.17 ^a^	0.20 ^ab^	0.18 ^ab^	0.009	0.031
C16:1 ^2^	3.30	2.93	2.79	3.04	2.83	0.526	0.728
C16:1x	0.51	0.48	0.43	0.46	0.47	0.043	0.420
C17:0	0.79	0.82	0.75	0.75	0.79	0.061	0.829
C17:1 ^2^	0.13	0.12	0.16	0.23	0.15	0.070	0.688
C17:0 *anteiso*	0.21	0.24	0.29	0.15	0.15	0.086	0.384
C18:0	15.5	15.2	15.0	15.2	15.7	1.02	0.677
C18:1 *trans*-9	0.21	0.22	0.21	0.19	0.19	0.018	0.371
C18:1 *trans*-11	1.04	0.84	0.91	0.92	1.11	0.092	0.058
C18:1 *trans*-12	0.21	0.20	0.22	0.20	0.22	0.017	0.636
C18:1 *cis*-9	33.3	33.8	32.7	32.9	30.0	1.94	0.319
C18:1 *cis*-11	1.42	1.46	1.45	1.51	1.48	0.101	0.924
C18:1 *cis*-12	0.21 ^a^	0.22 ^ab^	0.29 ^b^	0.21 ^a^	0.28 ^ab^	0.026	0.015
C18:1 *cis*-13	0.20	0.21	0.20	0.19	0.18	0.035	0.870
C18:2 *trans*-11, *cis*-15 ^2^	0.20	0.18	0.17	0.18	0.20	0.024	0.309
C18:2 *n–*6 *cis* ^2^	5.93	6.20	7.38	6.43	8.70	1.613	0.319
C18:3 *n–*6 ^2^	0.11	0.20	0.10	0.15	0.08	0.085	0.521
C18:3 *n–*3 ^2^	1.91	2.16	2.11	2.26	2.78	0.527	0.491
C18:2 *cis*-9, *trans*-11	0.32	0.27	0.29	0.27	0.33	0.028	0.310
C20:3 *n–*6	0.44	0.50	0.58	0.56	0.53	0.096	0.741
C20:4 *n–*6 ^2^	2.10	2.29	2.52	2.41	2.66	0.411	0.844
C20:5 *n–*3 ^2^	0.77	1.03	0.96	1.00	1.05	0.200	0.775
C22:4 *n–*6	0.19	0.19	0.21	0.20	0.21	0.027	0.884
C22:5 *n–*3 ^2^	1.31	1.49	1.55	1.47	1.57	0.232	0.900
C22:6 *n–*3 ^2^	0.20	0.25	0.22	0.21	0.21	0.068	0940
∑ Saturated FA	44.4	43.0	43.1	43.5	43.1	1.35	0.846
∑ Monounsaturated FA	41.8	41.7	40.5	41.0	38.2	2.24	0.496
∑ Polyunsaturated FA ^2^	13.8	15.2	16.4	15.5	18.7	3.08	0.630
∑ *n–*3 FA ^2^	4.36	5.13	5.02	5.13	5.84	0.973	0.765
∑ *n–*6 FA ^2^	8.8	9.5	10.8	9.8	12.3	2.14	0.519
*n–*6*/n–*3 FA ^2^	2.02 ^abc^	1.85^a^	2.15 ^c^	1.91 ^ab^	2.11^bc^	0.077	0.002
Atherogenicity index	0.59	0.54	0.55	0.56	0.54	0.088	0.762
Thrombogenicity index	1.03	0.96	0.96	0.97	0.93	0.080	0.812

SEM: standard error of the means. ^1^ Only fatty acids making up > 0.2 g/100 g are displayed, all others were considered as traces. ^2^ Data were transformed for statistical analysis but means of nontransformed data are presented in the table. ^a,b,c^ Means carrying different superscripts within variable are different at *p* < 0.05.

## Data Availability

The datasets used and analysed during the current study are available from the corresponding author on reasonable request.
